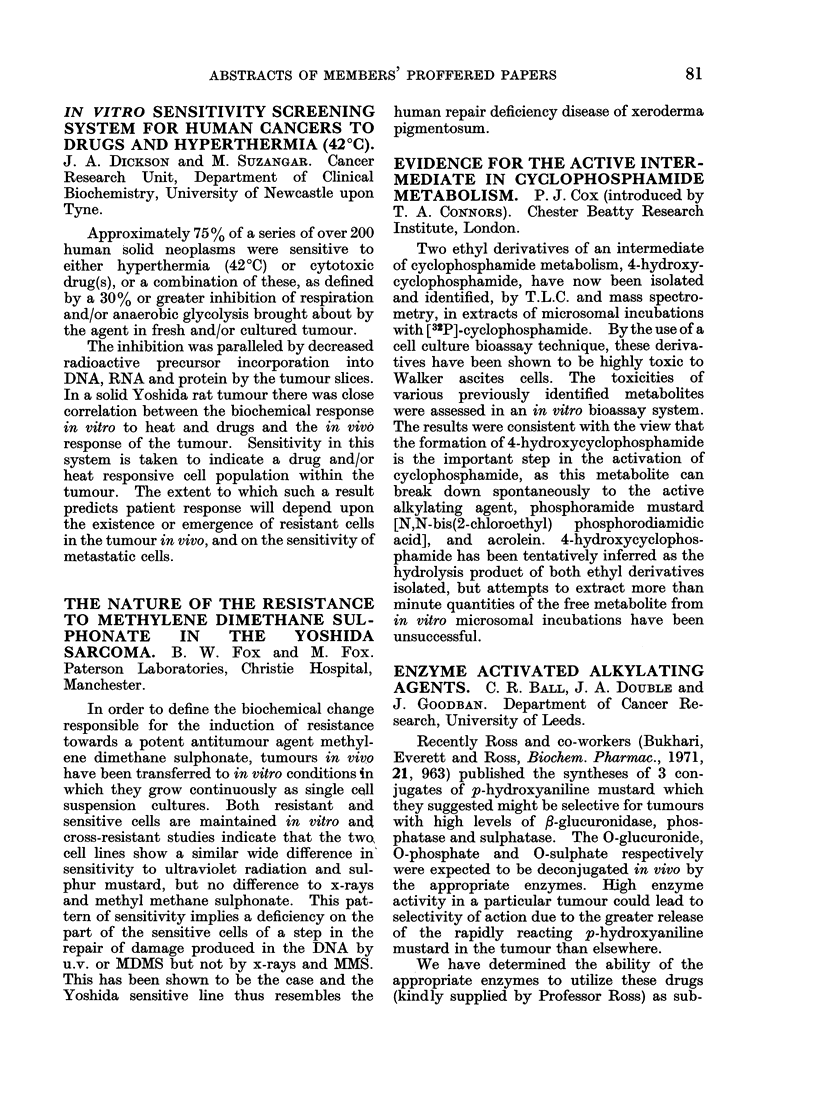# In vitro sensitivity screening system for human cancers to drugs and hypertermia (42 degrees C).

**DOI:** 10.1038/bjc.1973.89

**Published:** 1973-07

**Authors:** J. A. Dickson, M. Suzangar


**_IN VITRO_ SENSITIVITY SCREENING SYSTEM FOR HUMAN CANCERS TO DRUGS AND HYPERTHERMIA (42°C).** J. A. DICKSON and M. SUZANGAR. Cancer Research Unit, Department of Clinical Biochemistry, University of Newcastle upon Tyne.

Approximately 75% of a series of over 200 human solid neoplasms were sensitive to either hyperthermia (42°C) or cytotoxic drug(s), or a combination of these, as defined by a 30% or greater inhibition of respiration and/or anaerobic glycolysis brought about by the agent in fresh and/or cultured tumour.

The inhibition was paralleled by decreased radioactive precursor incorporation into DNA, RNA and protein by the tumour slices. In a solid Yoshida rat tumour there was close correlation between the biochemical response *in vitro* to heat and drugs and the *in vivo* response of the tumour. Sensitivity in this system is taken to indicate a drug and/or heat responsive cell population within the tumour. The extent to which such a result predicts patient response will depend upon the existence or emergence of resistant cells in the tumour *in vivo*, and on the sensitivity of metastatic cells.